# Elucidating genetic variability and population structure in *Venturia inaequalis* associated with apple scab diseaseusing SSR markers

**DOI:** 10.1371/journal.pone.0224300

**Published:** 2019-11-06

**Authors:** Sheikh Mansoor, Nazeer Ahmed, Vikas Sharma, Sumira Jan, Sajad Un Nabi, Javid I. Mir, Mudasir A. Mir, Khalid Z. Masoodi

**Affiliations:** 1 Division of Biochemistry, Sher-e-Kashmir University of Agricultural Sciences and Technology Jammu, Jammu and Kashmir, India; 2 Transcriptomics Laboratory, Division of Plant Biotechnology, Sher-e-Kashmir University of Agricultural Sciences and Technology (SKUAST-K), Shalimar, Srinagar, Jammu and Kashmir, India; 3 ICAR-Central Institute of Temperate Horticulture, Rangreth, Srinagar, Jammu and Kashmir, India; National Cheng Kung University, TAIWAN

## Abstract

Apple scab caused by *Venturia inaequalis* Cooke (Wint.) is one the important diseases of trade and industrial significance in apple. In present study variability studies in pathogen isolates were studied, which is one of the most important factors for devising management studies of scab disease in apple. Genetic diversity of 30 *Venturia inaequalis* isolates from 12 districts of two geographical distinct regions of Jammu and Kashmir was calculated based on the allele frequencies of 28 SSR markers and the internal transcribed spacer (ITS) region of the ribosomal DNA. The ITS based characterized sequences were submitted to NCBI GenBank and accession numbers were sanctioned. Dendrogram showed that all the accessions formed 2 main clusters with various degree of sub clustering within the clusters. Analysis based on SSR study reveals that the heterozygosity ranged from 0.0 and 0.5, with an average value of 0.39. The expected heterozygosis or gene diversity (He) ranged from 0.0 to 0.50 with an average of 0.40. The F_st_ value ranges from 0 to 0.6 with an average of 0.194. Diversity within each population (HS) values ranging from 0.26 to 0.33. Average differentiation among populations (GST) was 0.11 and populations were isolated by significant distance (r 2 = 0.50, P < 0.01). From the AMOVA analysis, 25% of variation was observed among population, 9% among individuals and 66% within individuals observed in the population. Structure analysis grouped isolates into two populations. Principle coordinate analysis explained variation of 36.6% in population 1, 14.30% in population 2 and 13.10% in population 3(Admixture) with 64.07% as overall cumulative percentage of variation. This indicates that extensive short-distance gene flow occurs in Kashmir region that dispersal over longer distances also appears to occur frequently enough to prevent differentiation due to genetic drift. Also it is evident that Jammu and Kashmir most likely has *V*. *inaequalis* subpopulations linked to diverse climatic conditions of the Jammu region compared to the mountainous inland Kashmir region. The results of present study would help to understand the genetic diversity of *V*. *inaequalis* from Jammu and Kashmir that would lead in the development of more effective management strategies and development of new resistant cultivars through marker-assisted selection.

## Introduction

Apple (*Malus × domestica* Borkh.) is most widely and commercially cultivated species in the genus Malus throughout temperate regions of world[1]. It is susceptible to number of diseases incited by fungi, bacteria, viruses, viroids and phytoplasmas[2]. Scab is very severe and among one of the mainsignificant diseasesall through the world in terms of economic losses in temperate regions with cool and moist climate during spring[3]. It ranks number one disease in terms of yield loss, which poses potential threat to apple industry[4]. This disease imposes a severe threat in commercial apple growing regions, due to premature fruit drop and unmarketable diseased fruits and results in losses up to 70%[5] or even complete crop loss is possible if prophylactic steps are not taken in the orchard for its management[6]. Scab disease is caused by *Venturia inaequalis* Cooke (Wint.) whichis an ascomycetous heterothallic and hemibiotrophicfungus. Earlier, this genus was included in the family *Venturiaceae*, order *Pleosporales*, according to its “Pleospora-type centrum and bitunicateasci” [7]. on the other hand, recent molecular phylogenetic analyses of Dothideomycetes, using both nuclear and mitochondrial gene regions, have indicated that the family Venturiaceae forms a well-supported monophyletic group separate from the Pleosporales[8, 9]. Thus, Zhang recently reordered Venturiaceae into Venturiales. It has a broad geographic dimension and is found in almost all apples growing areas. The fungus exists in two states i.e., saprophytic (sexual state *Venturia inaequalis* (Cke) and parasitic (asexual state *Spilocaeapomi* Fr)[3]. It overwinters as pesudothecia in regions with severe winter, whereas, conidia in dormant buds in regions with moderate winter [10]. In early spring, when temperature and moisture are suitable, Ascospores start maturing and are released forcibly in air[11].

One sexual and multiple asexual cycles, of this pathogen annually causes noteworthy variations in *Venturia inaequalis* population[12]. Recombination takes place by sexual reproductionwhich ultimately leads to high variation and diversity in fungi and also changes population genetic structure[13]. In devising the management strategies against the disease, important factor that is taken into contemplation is variationwithin pathogen population[14]. Detailed investigations about pathogens variation and population genetic structure in different geographical regions are required, which reflects the history as well as evolutionary potential of the pathogen [15] and also give an idea about centers of origin of this pathogen[16]. In wide range of organisms the ribosomal rRNA genes sequence investigation and the internal transcribed spacer (ITS) region is used as new tool in phylogenetic relationship studies [17]. As rRNA repeat develop slower as a result it is very handy for studying secluded related organisms[18]. Besides sequence divergence in ITS sequences, polymerase chain reaction (PCR) amplification length polymorphism in nuclear rDNA due to intron insertion has also been used to assess the extent of genetic variability within populations [19]. As per reports ITS based analysis is the best was to identify sub species than rbcL and matK[20]. Keeping in view the above background information, detailed investigations on the molecular characterization, genetic diversity and population structure of the *Venturia inaequalis* in numerousapple growing districts of Jammu and Kashmir was carried out.

## Materials and methods

### Collection of isolates

**Fig 1:**
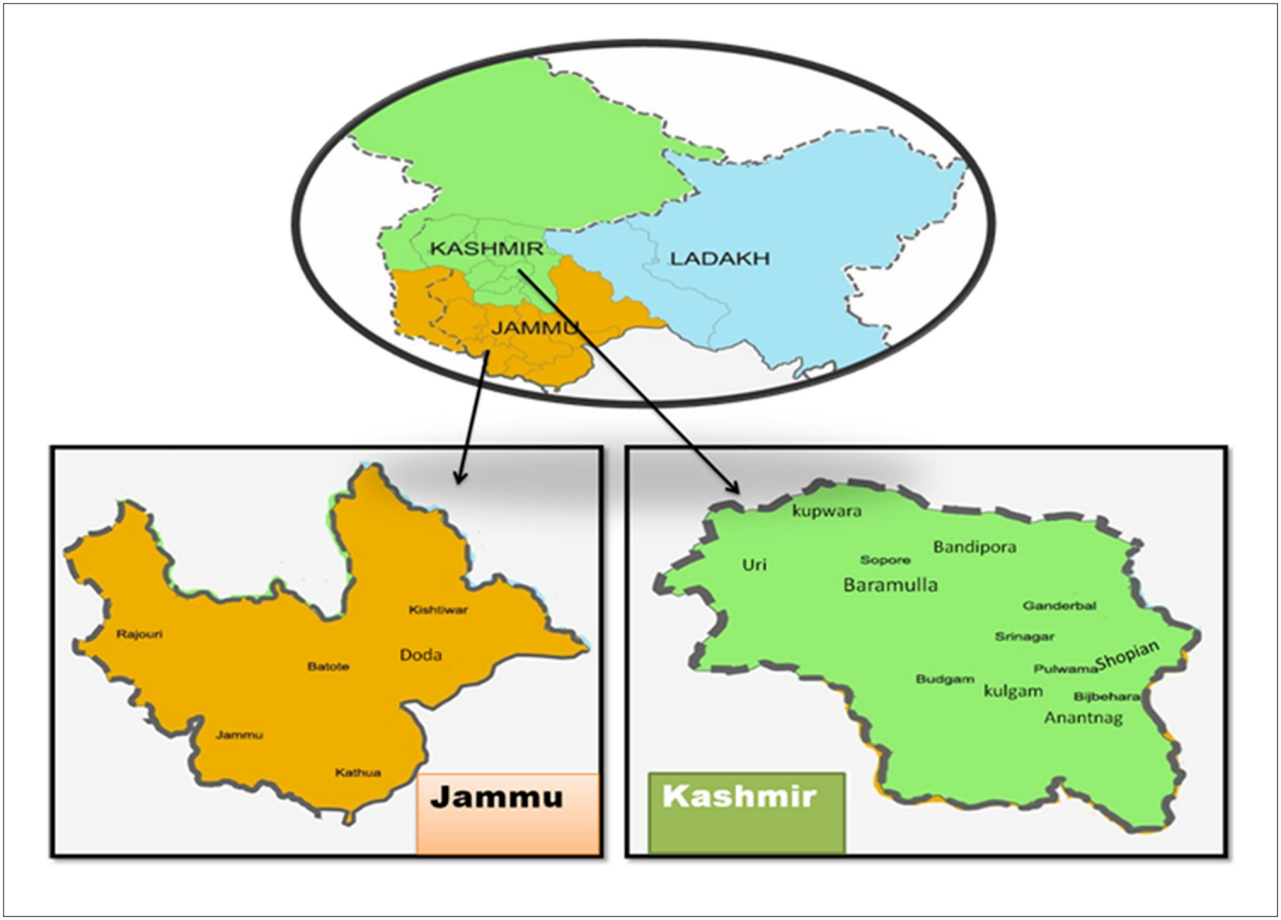
Two apple growing regions of Jammu and Kashmir and areas from where sample collection was carried out.

Samples were collected from 12 apple growing districts of Jammu and Kashmir India during the year 2017–18 as shown in Table 1. The locations sites from where samples were collected are provided in Fig 1. Diseases samples including only apple leaves with scab symptoms were collected from May to September 2018–19. Most of the cultivars were Red Delicious and Golden Delicious as these are most cultivated apple varieties grown in Jammu & Kashmir. Sampling was carried out from trees having at least two to three scab lesions on leaf. The samples were collected as part of thesis work and due permissions from farmers of the various orchards. The present study did not involve any endangered or protected species of the region.

**Table 1:** Samples collected from two regions of Jammu and Kashmir with sample code, geographic location, latitude, longitude and accession number provided by Genbank from various apple growing areas.

S.No	Sample Code	Location	District	Latitude	Longitude	Accession number
**Kashmir region isolates**						
1	M1	Trehgam	Kupwara	34.521°N	74.184°E	MK478885.1
2	M2	Bankoot	Bandipora	34.420°N	74.650°E	MK359025.1
3	M3	Char-i-sharief	Budgam	33.862°N	74.766°E	MK504436.1
4	M4	Syedpora	Shopian	33.72°N	74.83°E	MK478887.1
5	M5	Beerva	Budgam	34° 001°N	74.5953°E	MK359026.1
6	M6	Chadoora	Budgam	33.802°N	75.100°E	MK504428.1
7	M7	Batpora	Srinagar	34.936°N	74.464°E	MK504429.1
8	M8	Hajin	Bandipora	34.09°N	74.79°E	MK359032.1
9	M9	Yaripora	Kulgam	33.7°N	75.0°E	MK530205.1
10	M10	Handwara	Kupwara	34.40°N	74.28°E	MK359027.1
11	M11	Kakapora	Pulwama	33.88°N	74.92°E	MK359028.1
12	M12	Wakura	Ganderbal	34.05°N	74.47°E	MK367580.1
13	M13	Gutlibagh	Ganderbal	34.09°N	74.09°E	MK504434.1
14	M14	Naidkhai	Bandipora	34.09°N	74.79°E	MK359029.1
15	M15	Gantmulla	Baramulla	34.086°N	74.033°E	MK504430.1
16	M16	Pinjura	Shopian	33.72°N	74.83°E	MK504437.1
17	M17	Pattan	Baramulla	34.85°N	74.37°E	MK504431.1
18	M18	Sogam	Budgam	34.020°N	74.780°E	MK504435.1
19	M19	Mattan	Anantnag	33.701°N	75.285°E	MK359031.1
25	M25	Uri	Baramula	34.080°N	74.05°E	MK532035.1
**Jammu region isolates**						
20	M20	PranuBaderwah	Doda	32.58° N	75.538° E.	MK359030.1
21	M21	NalthiBaderwah	Doda	32.93°N	75.712°E	MK583539.1
22	M22	Doda	Doda	33.13°N	75.57°E	MK504432.1
23	M23	Kishtwar	Kishtwar	33.32°N	75.77°E	MK504433.1
24	M24	Tund	Kishtwar	33.32°N	75.77°E	MK532037.1
26	M26	Faisal Abad	Kishtwar	33.32°N	75.77°E	MK532036.1
27	M27	Padder	Kishtwar	33.13°N	75.09°E	MK532034.1
28	M28	Padder	Kishtwar	33.13°N	75.220°E	MK532033.1
29	M29	KotiBaderwah	Doda	33.145°N	75.547°E	MK532032.1
30	M30	Baderwah	Doda	33.13°N	75.57°E	MK532031.1

### Isolation, purification and identification of fungal cultures

The fungus from the infected samples was isolated and purified using monoconidial method by streaking out spores on plates containing 2% water agar, pure fungal cultures were obtained by transferring single germinated conidium on potato dextrose agarcontaining antibacterial chloromphenicol (50 μg/ ml) to avoid bacterial contaminations[21]. Total 30 cultures were identified by comparing with available literature[22] and maintained for further studies. The spores were also verified using compound microscope (Olympus) at different resolutions 4x to 40x.

### DNA extraction

Cultured fungal isolates in 100ml of potato dextrose broth was kept in incubator-shaker at 19°C for about 25–30 days under continuous dark. The mycelia harvested was blotted dry between the tissue layers and immediately frozen in liquid nitrogen. After freeze-drying, DNA was extracted using Fungal DNA isolation kit (GCC Biotech India Pvt. Ltd). The DNA was quantitatively and qualitatively checked using a Nanodropspectrophotometer (Themoscientific) and was further diluted to a workingconcentration of 30ng/μl and stored at -20°C for further use.

### PCR amplifications

#### ITS rDNA amplification

All 30 isolates were amplified using polymerase chain reaction (PCR) in a thermal cycler (Takara Japan) using 30ng of genomic DNA in a final volume of 25 μl per reaction. The universal ITS primers with ITS 1 as forward and ITS4 as reverse primerwere used for PCR amplification (White et al., 1991). ThePCR was performed in a 0.2-ml tube containing 0.5μM forward and reverse primer, 200 μM eachdNTP, 1 unit kappa Taqpolymerase and 1ul of genomic DNA in a 10xkappa buffer and 5 mM MgCl_2_. The PCR was normalized after repetitive cycles till optimal amplification was achieved and consists of 35 cycles involving initial denaturation step at 94°C for 5 min, followed by 94°C for 30 s, annealing at 53°C for 45 s, extension at 72°C for 1min and final extension at 72°C for 15min[23]. The PCR products were electrophoresed in 1% agarose gel in 0.5 X Tris-Borate-EDTA buffers (89 mMTris-HCl, 89 mM boric acid, 2.5 mMEDTAand pH 8.5) at 110V. For estimating amplicon size, 100 bp DNA molecular ladder was used (ABgene, UK) and electrophoresis was done for 1 hour[24]. The fragments were observed under UV lamp in gel-documentation (Bio Rad, Gel Doc XR system 170–8170).

#### SSR amplification

For diversity and structure analysis of selected fungal samples, 28 published SSR primer pairs were used Table 2 [11, 13]Conditions for PCR were initial denaturation at 94°C for 3 min, followed by 35 cycles of denaturation step at 94°C for 30 s, 45 s of annealing at 50–60°C, 1min of extension at 72°C, and a final extension of 15 min at 72°C which was performed in a 10 μl final volume containing 2 μl of 10X PCR buffer, 3 mMMgCl2, 0.5mMdNTP, 0.5μl of *Taq* DNA polymerase (kappa), 1μM of each primer and 1μl DNA template [23]. The amplified PCR products were resolved in 2.5% agarose gel at 110 V for 3 h. The bands amplified in different isolates using SSR primers in gel werevisualized using a Gel documentation system (Bio Rad, Gel Doc XR system 170–8170).

**Table 2:** List of 28 SSR primers with forward (F) and reverse(R) sequence, allele size and annealing temperature.

Locus	Primer sequence 5′–3′	Allele Size (bp)	Annealing Temp.
*Vitc1/2*	F:CTTACCTCTCACTTGCTAAC R:GTTCTGACAAGACTGTGTTG	173–241	58
*Vitc1/82*	F:ACTGTCTCTAGGCGAAAG R:ACTTGGAAGCTCGCTAAG	227–247	58
*Vitc1/130*	F:GATTGGTGACGCATGTGT R:GCTGGAGATTGCGTAGAC	132–152	58
*Vitc2/D*	F:GCTCCTTCTGGGTAAGA R:CTCTACATCTCATCCCATC	184–278	58
*Viaggt8/1*	F:GTGCGGAATATCCAAGTA R:CCAGACTCCTCTACTACAACC	188–196	58
*Vica9/152*	F:GCACCTGCTCTGTCTATCTC R:AAGGTTCAGGCACTGGAG	167–191	58
*Vitcca7/P*)	F:GAATACTTCCAAGTGCACAG R:GTGACGCGGATATGGTAC	192–224	58
*Vitg11/70*	F:GAAGAGGTTGGAGTGGTTG R:GAACCGAATCTGTACAGGAC	184–196	60
*Vica9/134*	F:ATACAGGAGTGAACAGCAGG R: ATACTGCTCACTTGCGTGC	228–236	60
*Vigtg10/95*	F: AGGTGTTGCTGTCTTGGAG R: CGATAGTGTCATTTCCAATCC	134–169	58
*Vicacg8/42*	F: TGTCAGCCACGCTAGAAG R: CACCGGACGAATCATGC	196–232	60
*Vigt8/146*	F: TGGAGAGAAGAGAAGAGTGG R: GGCAACATCCAATAATCG	128–134	60
*Vitc2/16*	F: ACATTGACGAAGACGAGC R: TACAATTGAGGCGTGTCC	147–165	58
*Viga3/Z*	F: ACGCCTCCTTACTTCTTG R: CCCTCGTATTACGTTCTC	87–97	58
*Viga7/116*	F: GCCTGGTTGTGGATCTGTC R: ATCCTGCTACATCGACCTTC	159–173	60
*Vitg9/99*	F: CCGTGTCGAGACCTAATATC R: TGGCTCTTCGTAAGTCCTTC	155–167	58
*Vica9/X*	F: TCGCGCATCACTATCTACAC R: AGACAGGAATGTGGTGGAAG	225–239	58
*Vica10/154*	F: CCTCCTTCCTATTACTCTCG R: CTGAAGCGAACCTATGTCC	108–172	58
*ViaacS10*	F: ATTCCAAGCCTTACAACACCC R: TCCACTTCACCCATCGTTC	180–186	58
*Vigt10/ε*	F: GCAGTGCAGGAATAGTAAGG R: GCTGTGATACCAGAGAACGA	171–173	60
*Vitg9/129*	F: CTAATTCAACTCGCTGCGTC R: TTTCAGCCAGCTAACCTAGG	267–285	58
1tc1a	F: TCGAGATCCTCAAACTTCCTT R: TTTTAACTGTGCGGCCTG	109–187	54
1tc1b	F: CGATTGGGGATATGAAGACTT R: TTAGTAATCAAATCGCACCCA	149–210	54
1tc1g	F:TCACTCAACAATACAGTTTCTTAG R: TTTCACGGTAGCGATAGGAG	111–185	57
1aac3b	F: AGCGCTAGGTCGTGAAATC R: TTTCTGAAGTGTGTGGGACAT	118–174	55
1aac4b	F: GGTGAGGAGGGAGACGAG R: CATCACGCCCCTATCAAAC	166–177	58
1aac4f	F: CTTGACAGACCACAGCGAC R: CTGACTGAGAGTGGCATCG	96–116	58
1aac4h	F: TCGTTCATCGTTCGTTTTTTCG R:AATAGTGCGTACCCATATATCCA	198–201	56

#### Sequencing, nucleotide alignment and phylogenetic analysis

Amplified PCR products were sequenced at Agri Genome Labs (Infopark Road, Kakkanad, Kerala, India). Primers for the sequencing PCR product were the same as for the PCR amplification. The sequences of PCR products were assembled using DNA baser V.4 program to produce complete contig. These were further aligned using CLUSTAL W method of Bio-Edit software and aligned sequences were deposited in NCBI GenBank. A database search of homologous sequences was performed by BLAST analysis at NCBI (http://ncbi.nlm.nih.gov/BLAST). The sequence generated from the present study and reference strain sequences retrieved from GenBank were used to construct phylogeny by neighbour joining method with 1000 replications for each bootstrap value using MEGA 7.0 software version [25]. The other species of *Venturia pirina and nashicola* were also included in phylogeny to separate *Venturia inaequalis* from these species. For validation of results, an out group non-fungal pathogen *Pseudomonas syringae* was selected.

#### Statistical analysis

Analysis was carried out using POPGENE for gene frequency, allele number, effective allele number, polymorphic loci, gene diversity, Shannon index, gene flow, genetic distance. The GenAlEx version 6.5 for distance-based analysis like AMOVA (Analysis of molecular variance), and PCoA. (Principle coordinate analysis)[26, 27]. The scoring was done as base pair scoring and binary scoring in which bands were scored as ‘1’ (for presence) and ‘0’ (absence) [28]. Index of association rd statistics was applied to examine associations of alleles among different loci [29, 30], which is a comprehensive measure of multilocus linkage disequilibrium[30]. DARwin software version 5.0.158 was used in phylogenetic analysis[31]. Population structureand individual clustering (K) was done by means of Structure software ver. 2.3.4 [32], ΔKmethod[33] was applied to best estimate K, and was computed using Structure Harvester ver. 0.56.3[34, 35].

## Results

### Morphological identification

**Fig 2:**
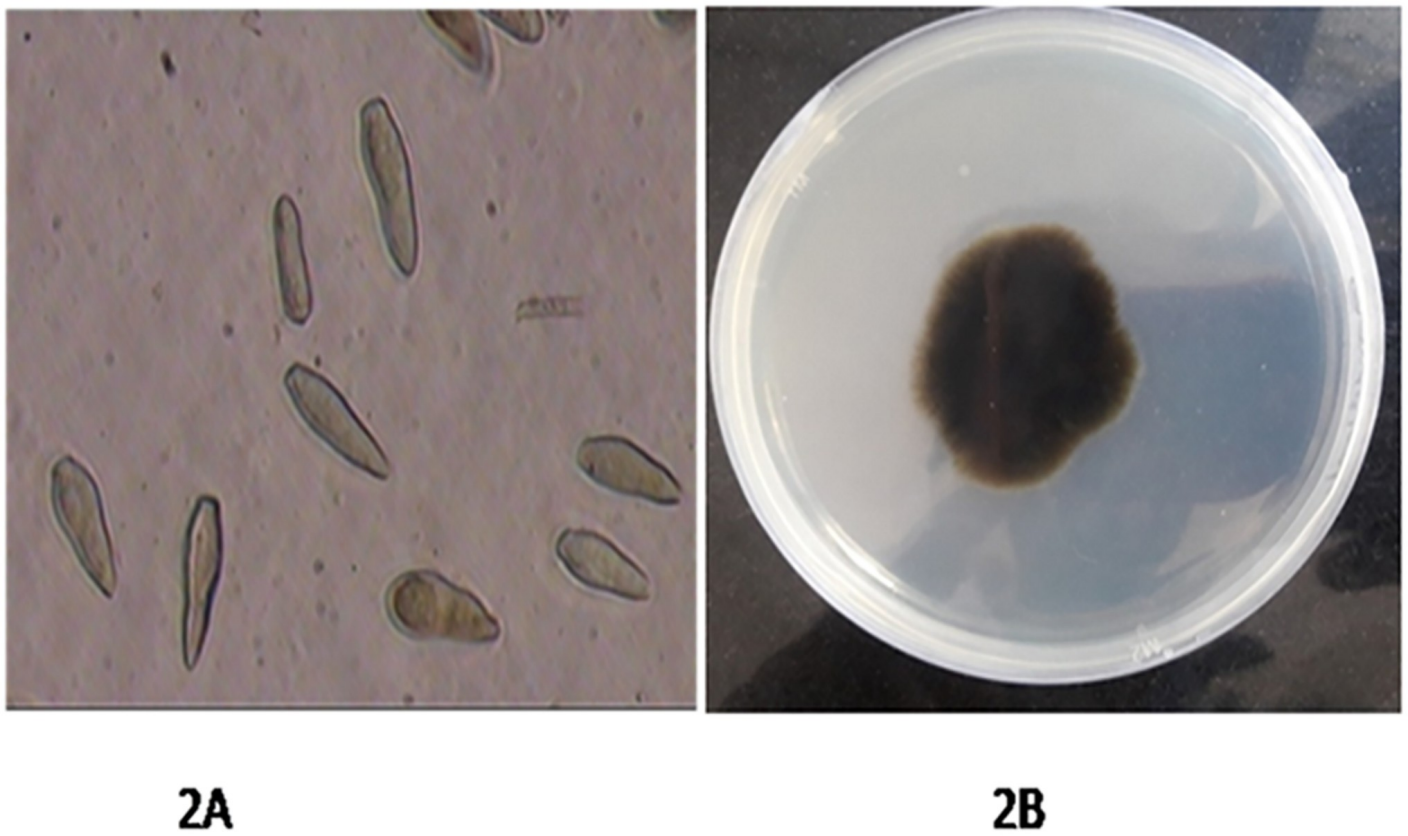
The spores verified under compound microscope (Olympus) at 10x to 40x. 2A. Conidia observed under 40 x resolutions. 2B. Pure culture of *Venturia inaequalis*.

Identification based on morphological characters from fungal culture (Fig 2a) revealed that the conidia are single-celled, uninucleate and narrower at one end than the other (Fig 2b). In mass, conidia appear brown or olive, but they are lighter when viewed individually under the microscope. Conidia ranges from 6 to12 μm wide and 12 to 22 μm long and are produced by specialized short hyphae called conidiophore. The characters observed were similar to those described by [22] for *Venturia inaequalis*.

### Molecular characterization

**Fig 3:**
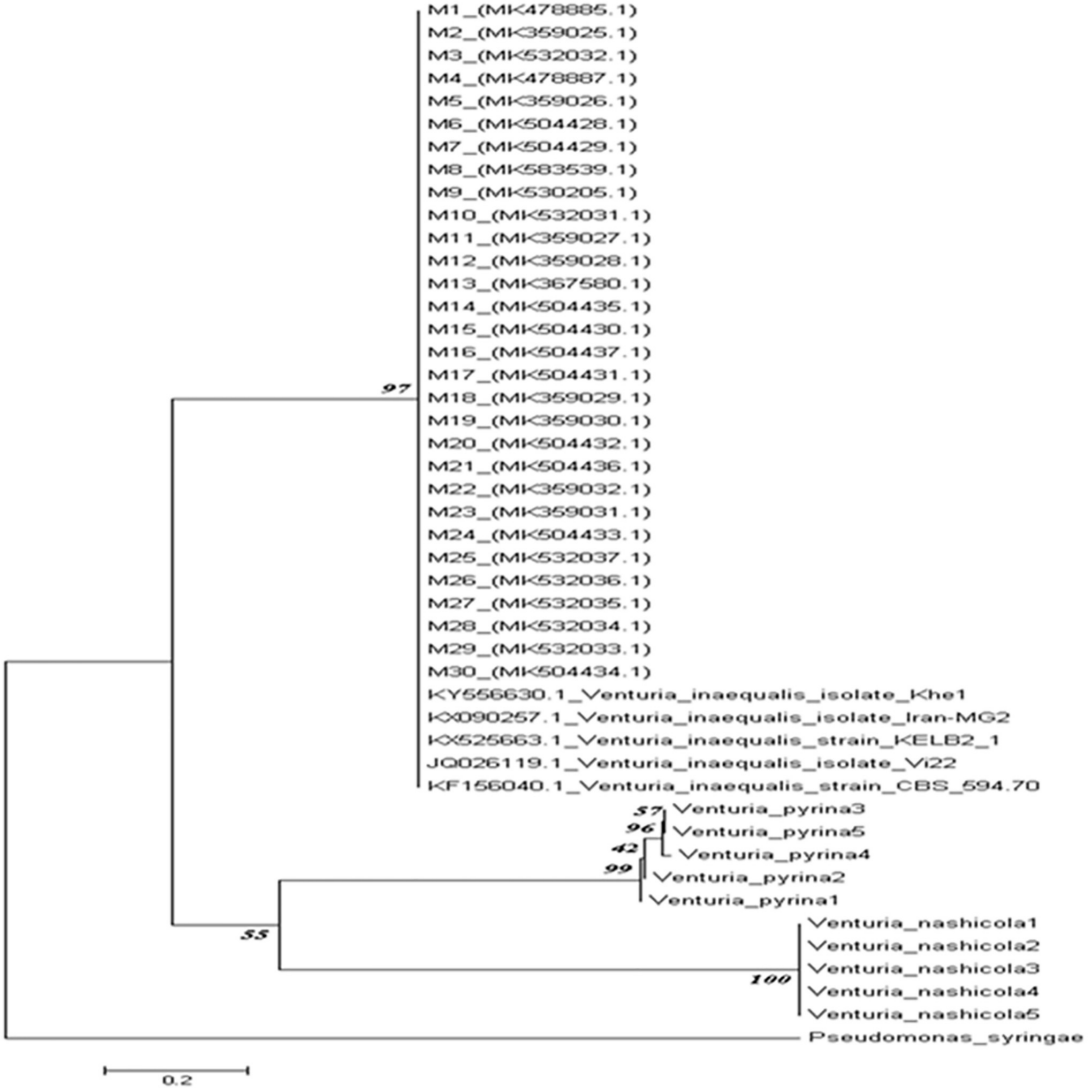
Phylogenetic relationship of *Venturia inaequalis* using the internaltranscribed spacer (ITS) region gene nucleotide sequence alignment with Bootstrap values supporting the branches are shown at nodes; branch lengthsare proportional to divergence.

The ITS based primers amplified ~550bp ampliconproducts after sequencing were run for BLASTn and all obtained sequences showed 96%-98% sequence homology with *Venturia inaequalis* GenBank submitted sequences. Sequences were submitted to NCBI GenBank and accession numbers were received (Table 1). Phylogenetic analysis revealed that our isolates clustered along with other submitted *Venturia inaequalis* isolates in GenBank. The sequences of other isolates *Venturia pirina* and *Venturia nashicola* formed separate subclusters. *Pseudomonas syringae* formed a different cluster (outgroup) in phylogeny (Fig 3). During present study the molecular characterization using ITS ribotyping of 30 isolates collected from two regions of J&K showed sequence homology with isolates reported from different regions of worlds particularly Iran (Khe1) and (MG2), South Africa (KELB2), India (Vi22) and Netherland (CBS).

### SSR genotyping and genetic diversity analysis

In total 30 *Venturia inaequalis* isolates were genotyped using 28 SSR markers. The results obtained through POPGENE software analysis for genetic diversity parameters are presented in (Table 3). The number of alleles per marker varied from 1 to 15 with an average of 4 per locus detecting the highest number of 15 alleles from Vitc1/2 and Vitc1/130 in the set of 30 isolates. The mean PIC value was found to be 0.073 with minimum value of 0.014 (Vitc1/130) and maximum of 0.195 (Vitg11/70). The average heterozygosity (Ave_Het) ranged between 0.0 and 0.5, with an average of 0.39. The expected heterozygosity or gene diversity (He) ranged from 0.0 to 0.50 with an average of 0.40. The effective number of alleles (Ne) ranged from 1.0 to 2.0 with an average of 1.74. Shannon index (I) an estimate of diversity ranged from 0 to 0.6 with an average of 0.57. The Fst value ranges from 0 to 0.6 with an average of 0.194.

**Table 3:** Summary of the genetic analysis of *Venturia inaequalis* isolates with 28 SSR loci.

Locus	Ne	I	Ob_He	Exp_Ho	Exp_He	Nei	Obs_Hom	Ave_Het	Pic	Fst
*Vitc1/2*	1.25	0.36	0.23	0.79	0.20	0.20	0.76	0.20	0.20	0.15
*Vitc1/82*	1.18	0.28	0.16	0.84	0.15	0.15	0.83	0.15	0.15	0.08
*Vitc1/130*	1.92	0.67	0.66	0.51	0.48	0.48	0.33	0.48	0.10	0.32
*Vitc2/D*	1.38	0.45	0.33	0.71	0.28	0.27	0.66	0.27	0.25	0.21
*Viaggt8/1*	1.99	0.69	0.83	0.49	0.50	0.49	0.16	0.49	0.16	0.64
*Vica9/152*	1.99	0.69	0.86	0.49	0.50	0.49	0.13	0.49	0.32	0.07
*Vitcca7/P*	2.00	0.69	1.00	0.49	0.50	0.50	0.00	0.50	0.52	0.00
*Vitg11/70*	1.86	0.65	0.73	0.52	0.47	0.46	0.26	0.46	0.20	0.12
*Vica9/134*	1.94	0.67	0.76	0.50	0.49	0.48	0.23	0.48	0.16	0.00
*Vigtg10/95*	1.86	0.65	0.73	0.52	0.47	0.46	0.26	0.46	0.07	0.24
*Vicacg8/42*	1.99	0.69	0.96	0.49	0.50	0.49	0.03	0.49	0.16	0.00
*Vigt8/146*	1.94	0.67	0.43	0.50	0.49	0.48	0.56	0.48	0.16	0.00
*Vitc2/16*	1.98	0.68	0.63	0.49	0.50	0.49	0.36	0.49	0.16	0.15
*Viga3/Z*	1.14	0.24	0.13	0.87	0.12	0.12	0.86	0.12	0.16	0.00
*Viga7/116*	1.96	0.68	0.86	0.50	0.49	0.49	0.13	0.49	0.16	0.00
*Vitg9/99*	1.99	0.69	0.96	0.49	0.50	0.49	0.03	0.49	0.16	0.00
*Vica9/X*	1.94	0.67	0.83	0.50	0.49	0.48	0.16	0.48	0.16	0.04
*Vica10/154*	2.00	0.69	1.00	0.49	0.54	0.50	0.00	0.50	0.16	0.00
*ViaacS10*	1.96	0.68	0.86	0.50	0.48	0.49	0.13	0.49	0.30	0.00
*Vigt10/ε*	1.99	0.69	0.96	0.49	0.50	0.49	0.03	0.49	0.10	0.00
*Vitg9/129*	1.64	0.57	0.53	0.60	0.39	0.39	0.46	0.39	0.25	0.00
1tc1a	1.00	0.00	0.00	1.00	0.00	0.00	1.00	0.00	0.15	0.00
1tc1b	2.00	0.69	1.00	0.49	0.50	0.50	0.00	0.50	0.12	0.00
1tc1g	1.30	0.39	0.26	0.76	0.23	0.23	0.73	0.23	0.10	0.00
1aac3b	1.76	0.62	0.63	0.55	0.44	0.43	0.36	0.43	0.13	0.00
1aac4b	1.86	0.65	0.73	0.52	0.47	0.46	0.26	0.46	0.15	0.00
1aac4f	1.06	0.14	0.06	0.93	0.06	0.06	0.93	0.06	0.10	0.18
1aac4h	1.96	0.68	0.73	0.50	0.49	0.49	0.26	0.49	0.15	0.00
**Mean**	**1.74**	**0.57**	**0.64**	**0.59**	**0.40**	**0.39**	**0.35**	**0.39**	**0.18**	**0.19**
**Standard deviation**	**0.34**	**0.19**	**0.31**	**0.15**	**0.15**	**0.15**	**0.31**	**0.15**	**0.08**	**0.03**

(Exp_Ho) Expected homozygosty (Exp_he) heterozygosity were computed using Levene (1949), (Nei) Nei’s (1973) Na = Observed number of alleles, Ne = Effective number of alleles [Kimura and Crow (1964)], I = Shannon’s Information index [Lewontin (1972)], (Ob_He) observed heterozygosity, (Ob_Ho) homozygosity, Fst = genetic differentiation.

### Cluster analysis

**Fig 4:**
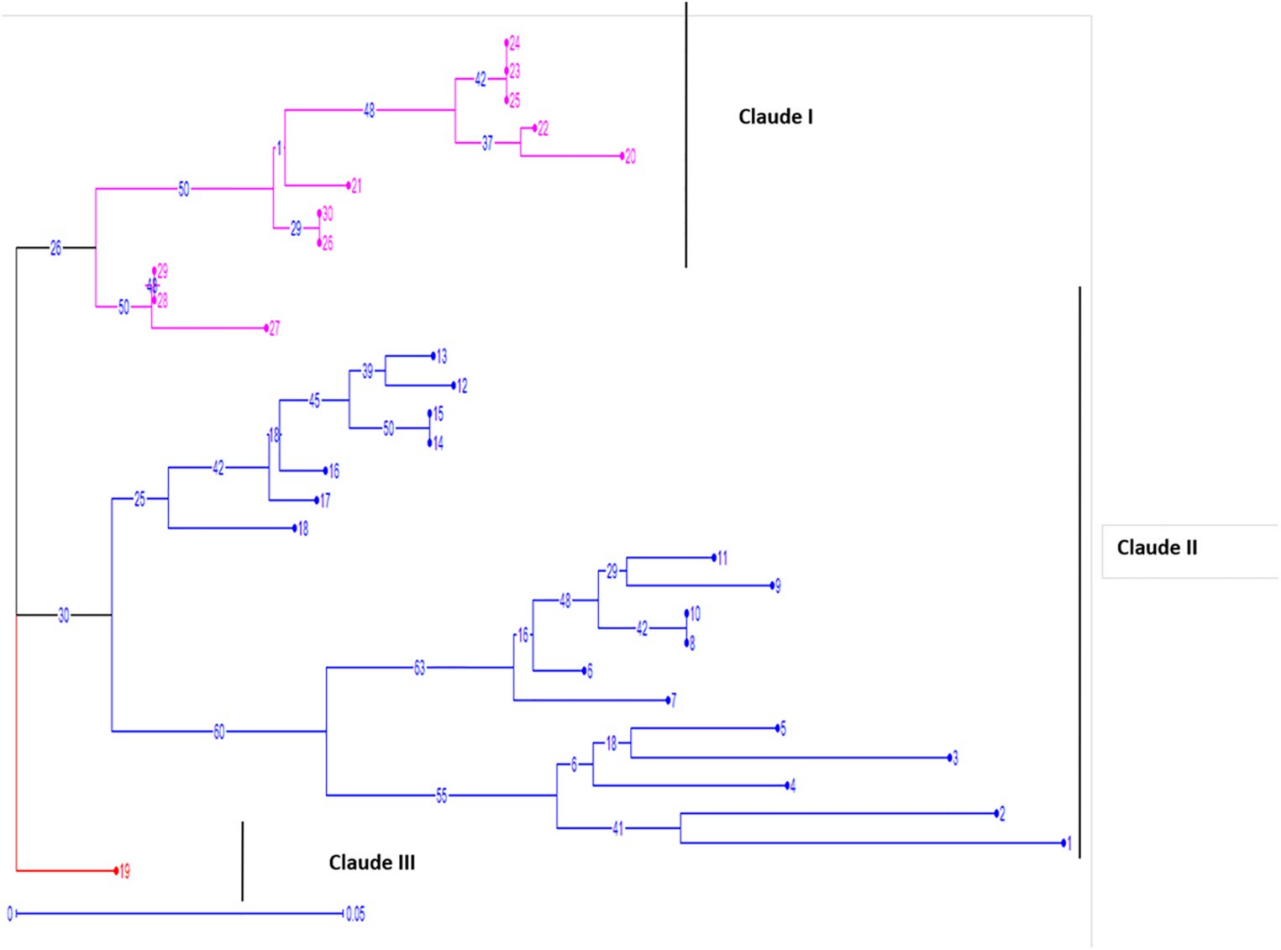
The cluster analysis (dendrogram) of 30 isolates revealing diversity within *Venturia inaequalis* populations. The bootstrap values are given on the nodes.

The cluster analysis of 30 isolates revealed a high genotypic diversity within *Venturia inaequalis* populations. Three major clusters I, II, III were obtained using neighbour joining method in Darwin 5.0 software using SSR scoring data. The cluster I accommodated 11 isolates (M20 to M30), cluster IIcontained 18 isolates (M1to M18) and Cluster IIIincluded only 1 isolate (M19). Both The Cluster I and II were further subclustered into two subclusters (Fig 4). The isolates could be grouped into separate clusters on the basis of geographical distribution as shown in Table 1.

### Population structure

**Fig 5:**
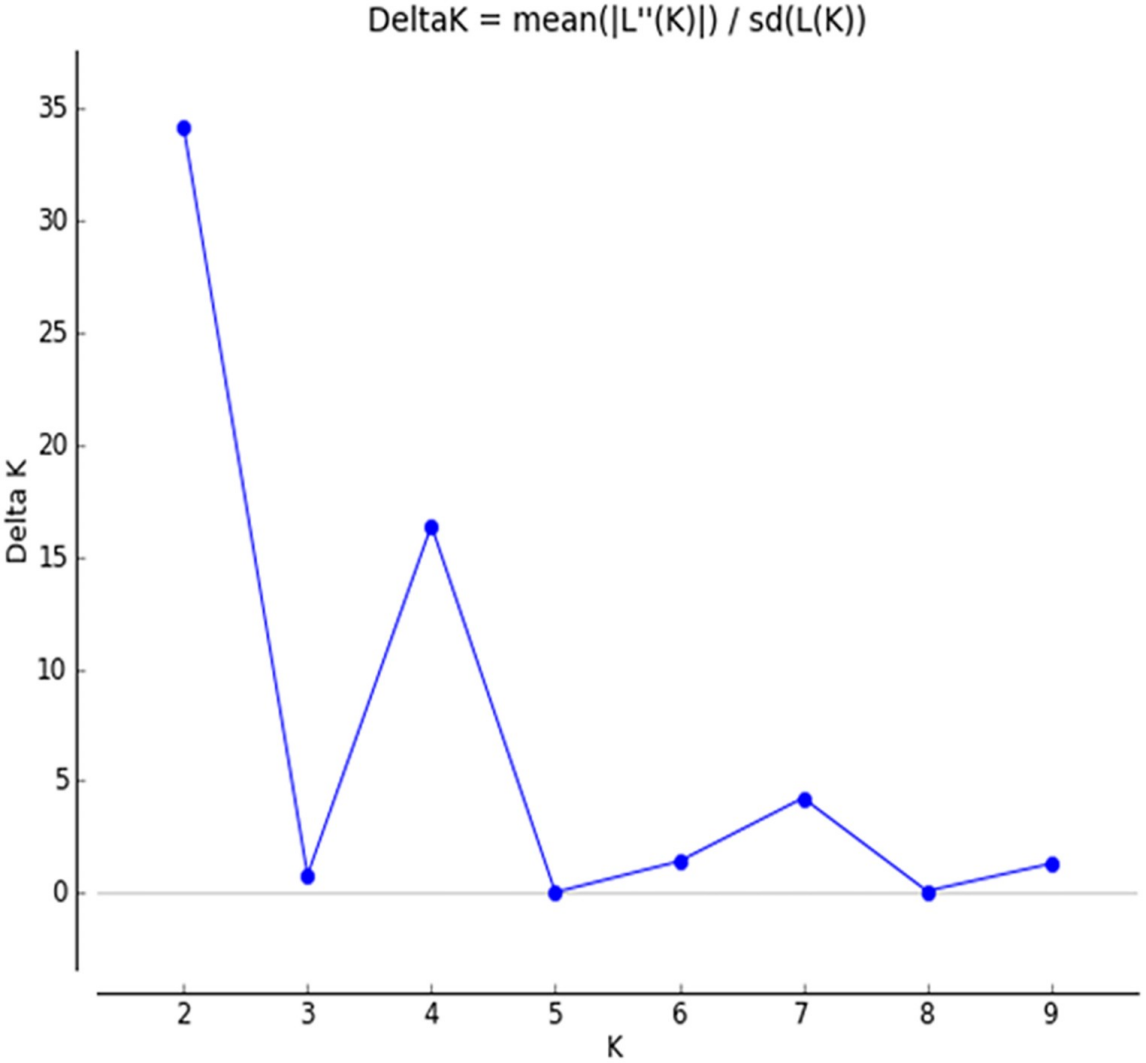
ΔK peak value of 34.6 among the assumed K.

**Fig 6:**
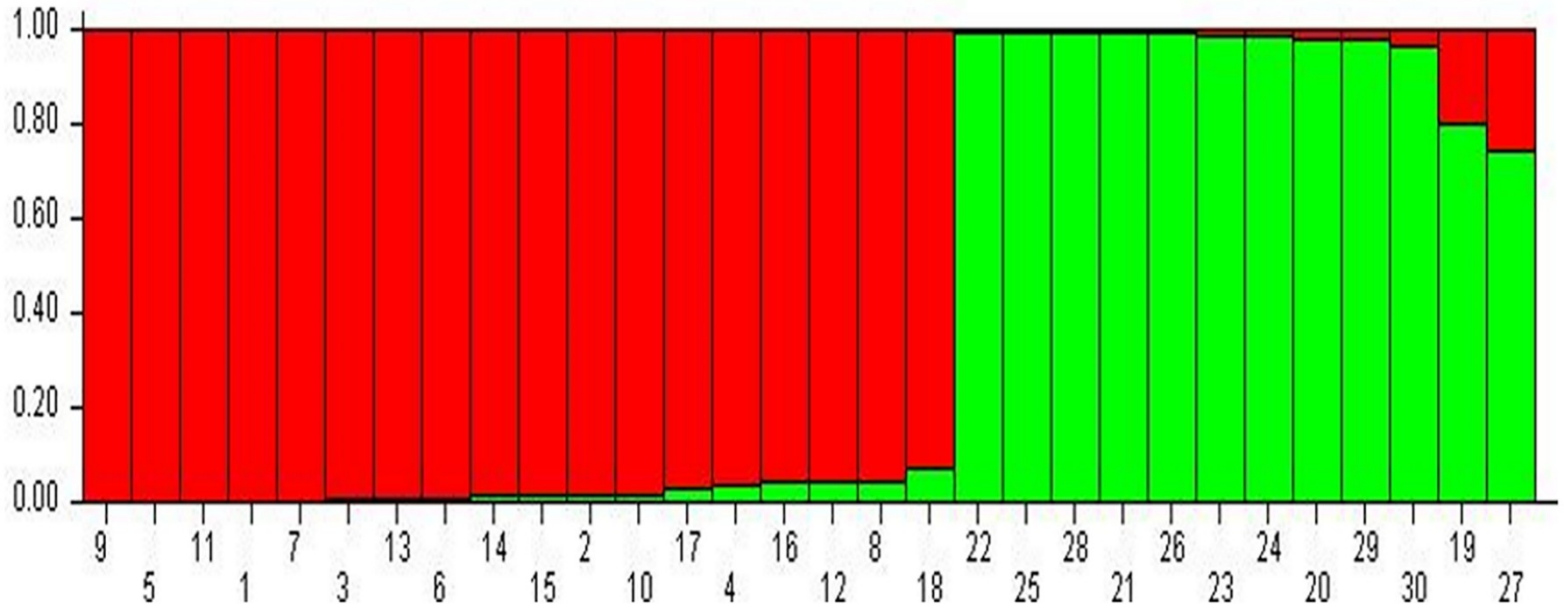
Population distribution using STRUCTURE analysis software, the isolates were grouped into two major populations with a small admixture (19 and 27 only).

Structure analysis revealed that isolates of *V*. *inaequalis* collected from different places Jammu and Kashmir were grouped into two major populations. The assumed values of probable sub-populations (K) were ascertained by choosing higher ΔK value, with respect to the number of clusters inferred by Structure [33]. As per the Evano table output, (S1 Table) the K = 2 was observed to be the best due to high ΔK peak value of 34.6 among the assumed K (Fig 5). Isolates from Jammu region having same latitude in J&K geographical map were grouped as subpopulation 1. Similarly, isolates from Kashmir region having same latitude in J&K geographical map were grouped subpopulation 2 with two admixtures (M19 & M27) (Fig 6). Moreover, STRUCTURE analysis grouped 2 individuals (6.6% of the total isolates) with a Q admixture proportion to the second cluster with the probability of 0.2 and 0.8, suggesting a substantial level of gene flow between the two clusters. Population 1 contains isolates from 1–18, while as population 2 comprised of isolates from 20–26, 28, 29 & 30. Two isolates (i.e. 19 &27) fall as admixture minimally.

### Analysis of molecular variance (AMOVA)

**Fig 7:**
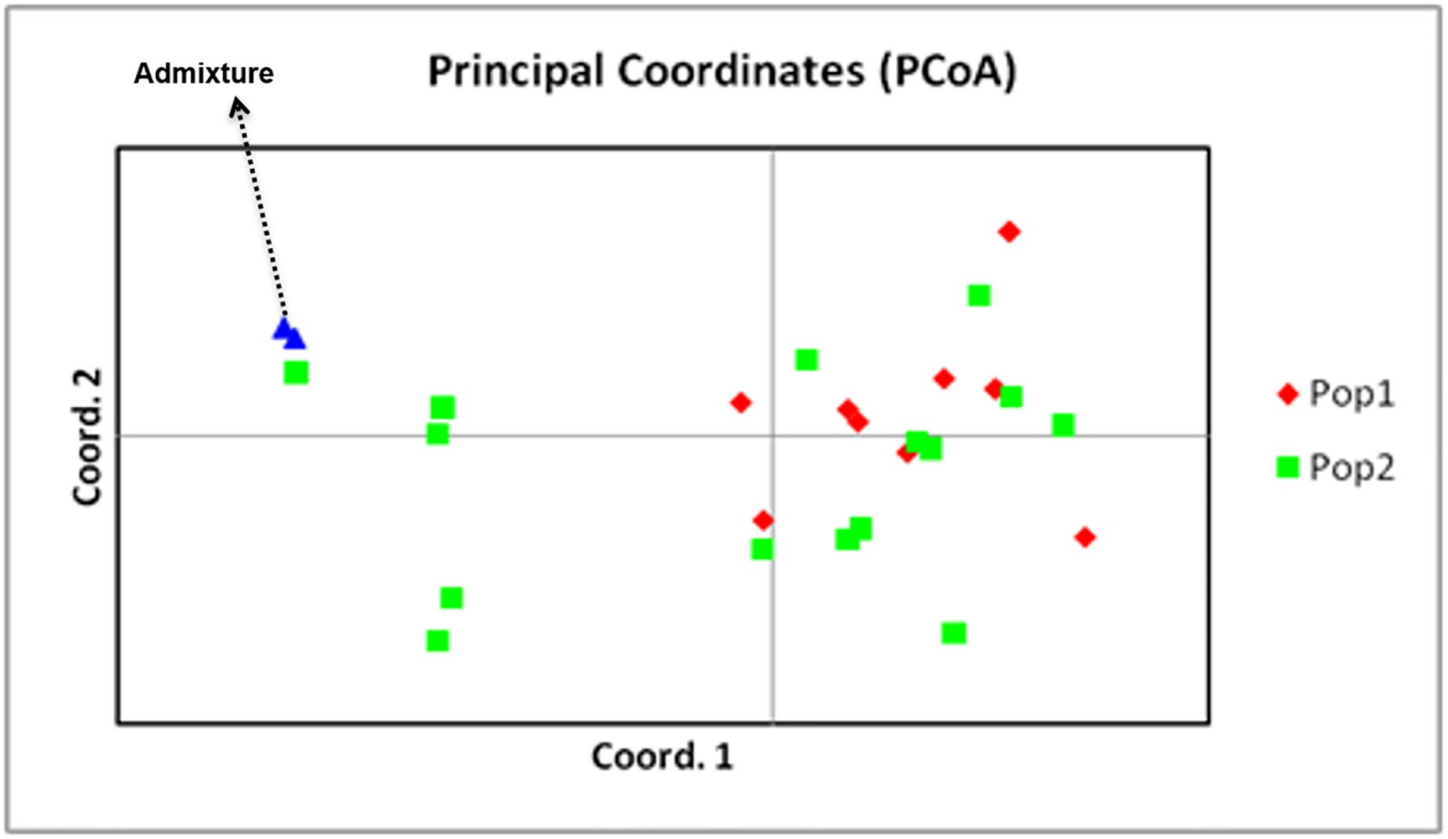
Principal coordinate analysis showing the clustering of two *Venturia inaequalis* populations and admixture population from different apple growing regions in the Jammu and Kashmir with population 1 (36.6%), population 2 (14.30%) and admixture (13.10%).

The two populations along with admixture isolates generated from structure analysis were analyzed for genetic variation among and within populations using AMOVA (Table 4). However, in population 3 sample sizes is less than 5which cannot be considered as a population. From the analysis, 25% of variation was observed among population, 9% among individuals and 66% within individuals observed in the population. Wright’s F statistic was estimated to determine deviation of Hardy-Weinberg expectation in the population. The F_is_ for all the 28 marker loci was 0.126, while F_it_ was 0.343 across the clusters. Pair wise F_st_ values showed significant differentiationamong all the pairs of sub-populations ranging from 0.248 to 0.881 suggesting that all the threegroups were significantly different from each other. The F_st_ values and their distribution pattern show clear differentiation of sub populations from each other. This result was also validated by the principal coordinate analysis (PCoA), (Fig 7) where co-ordinate 1 and 2explained variation of 36.6% in population 1, 14.30% in population 2 and 13.10% in population 3 and overall cumulative percentage of variation of 64.07%.

**Table 4:** Genetic variation among and within populations using AMOVA.

Source	Df	SS	MS	Est. Var.	%
Among Pops	2	21.720	10.860	0.554	25%
Among Indiv	27	50.980	1.888	0.211	9%
Within Indiv	30	44.000	1.467	1.467	66%
Total	59	116.700		2.231	100%
**F-Statistics**	**Value**	**P**			
**F**_**st**_	0.248	0.001			
**F**_**is**_	0.126	0.040			
**F**_**it**_	0.343	0.001			

Df-Degree of freedom, SS Sum of squares, MS Mean sum of squares, Est. Var-Estimated variance

## Discussion

Apples are grown in high altitude areas of India particularly in J&K[36]. It is the primary cultivated crop in J&K because though the climate conditions are distinct (sub-temperate to true temperate), they are very suitable for the cultivation of apples[37]. This crop suffers huge losses both quantitatively and qualitatively due to frequent epidemics of scab disease [23, 38], which is caused by *V*. *inaequalis*. The molecular characterization elucidates the genetic diversity among the isolates and for better resistance against any pathogen, the diversity must be known and accordingly resistant varieties can be developed. Hence in present study the molecular characterization of *V*. *inaequalis* was undertaken in order to provide better management strategies for this disease in the form of resistance and cisgenic breeding approaches.

We used ITS ribotyping of 30 isolates collected from two distinct regions. The noncoding ribosomal DNA ITS sequences doesn’t change more rapidly than the coding sequences and may diverge between species and populations [18]. AnalyzingITS regions has become one of the primary methods for identification and characterization of a fungal strain or species [39, 40]. We observed sequence homology with isolates reported from different regions of the world particularly Iran, South Africa, Netherlands, and Canada. As expected, after phylogenetic analysis of 45 *Venturia* sequences (35 *V*. *inaequalis*, 5 *V*. *pirina*, *and 5 V*. *nashicola)*, two clades emerged: one with all the *V*. *inaequalis* sequences and another with the *V*. *pirina* and *V*. *nashicola* sequences that further separated into two subclades. Bilal *et al*[21] had similar results. The current study identified taxonomic relationships or differences between 30 *V*. *inaequalis* isolates, which can help to identify characteristics such as resistance or susceptibility toward a particular anti-fungal agent.

Microsatellites or SSRs are very useful markers for population genetics analysis because of their high specificity, polymorphism, and reproducibility. These are major advantages of using SSR markers over Random Amplification of Polymorphic DNA. The SSR markers used in this study were highly variable and results generally corresponded with previous population genetics studies conducted in Europe. However, in this study the markers1tc1a, 1tc1b, 1tc1g, 1aac3b, 1aac4b, 1aac4f, and 1aac4h showed only one allele compared to eight to ten alleles reported previously[41, 42]. The outcomes of this study along with prior reports[4, 11, 13, 21]confirm the continuation of high variability in *V*. *inaequalis*.

The distance between the Jammu region and the Kashmir region where we collected samples is approximately 400 km and allowed us to collect *V*. *inaequalis* isolates from geographically and topologically distinct regions. We observed a high level of diversity, which could be expected due to the climate differences. Xu *et al*[43]also observed remarkable variability between and within *V*. *inaequalis* isolates obtained from dissimilar apple cultivars in a solo apple orchard. This was credited to assortment pressure applied by diverse cultivars. Another possible explanation of variation in diversity is sexual recombination.

The isolates in this study shared a high percentage of identical alleles, indicating considerable gene flow among all isolates of *V*. *inaequalis* populations in J&K. Asmost of the apple cultivation area in Jammu and Kashmir is dominated by single cultivar Red Delicious and no resistant variety is under cultivation yet, so there is no selection pressure on pathogen to bring some change, hence this could be the reason having high percentage of identical allelesamong *V*. *inaequalis* populations. The movement of the *V*. *inaequalis* from one place to other can be through planting material, high speed winds, but the planting material was one of the most important factors of introduction of *V*. *inaequalis* to India. This was also observed in the magnitude of migration between the regions. The Kashmir region seems to have the highest migration rate towards it and the lowest away from it. Migration towards the Jammu region was the lowest, indicating that this region is isolated, probably due to warmer winter temperatures. Overall the migration results indicated the possible free movement of the pathogen between the regions. The present study undoubtedly shows that there is high diversity of *V*. *inaequalis* in the Kashmir valley and reveals that the larger part of variability existed within the individuals.

Structure analyses divided the isolates into two populations (K = 2) with a clear differentiation between the two apple-growing regions. Pathogen distance seems to be the most significant factor in steep gene flow because it explains 50% of the variation among the *V*. *inaequalis* isolates. Pair-wise F_st_ values, ranging from 0.248 to 0.881 showed noteworthy demarcation between the subpopulations. This signifies that the two groups are notably dissimilar from one another. This outcome is also validated by the principal coordinate analysis, whichdistributed the isolates into two major populations with one admixture (two isolates). The admixture is of smallsize, hence can’t be considered as a population.

## Conclusion

The genetic variation and population structure of scab causing *V*. *inaequalis* from different apple growing regions in Jammu and Kashmir shows significant levels of genetic variation within the populations in the similar fashion as observed in other *V*. *inaequalis* population’s studies conducted in Europe and elsewhere. Results indicated that gene flow between regions is occurring and has significant implications for the apple industry if fungicide resistant strains move between regions. Based on ITS sequencing, a database can be maintained to list out the sequence-based isolation of various fungal strains or species. In order to control such menace caused by scab, proper prediction and forecasting systems are need of the hour to prevent apple scab disease well in advance and also understanding the host pathogen interaction, which can provide new insights for effective management of this disease. Cisgenesis can be one of the approaches for introgression of resistance gene through biotechnological intervention under control of its own regulatory sequences from same species or related species which can also maintain the original cultivar characteristics.

## Supporting information

S1 Table (DOCX)
