# Supplementary material for: Elucidating genetic variability and population structure in Venturia inaequalis associated with apple scab diseaseusing SSR markers
Source: PLoS One. 2019 Nov 6;14(11):e0224300. doi: 10.1371/journal.pone.0224300 (PMC6834240; doi:10.1371/journal.pone.0224300)
Supplement: S1 Table — (DOCX) [file pone.0224300.s001.docx]

S1 Table

| **k** | **lnpk** | **stdevlnpk** | **Ln’k** | **Delta K** |
| --- | --- | --- | --- | --- |
| 1 | 0.667 | 0.960 | NA | 0.00 |
| 2 | 0.233 | 1.429 | 72.833 | 34.138 |
| 3 | 0.200 | 15.616 | 24.033 | 0.806 |
